# Myalgic Encephalomyelitis/Chronic Fatigue Syndrome: Organic Disease or Psychosomatic Illness? A Re-Examination of the Royal Free Epidemic of 1955

**DOI:** 10.3390/medicina57010012

**Published:** 2020-12-26

**Authors:** Rosemary Underhill, Rosemarie Baillod

**Affiliations:** 1 Independent Researcher, 7 Avenue De La Mer #506, Palm Coast, FL 32137, USA; 2 Independent Researcher, 2, Wold Rd. Hull, Yorkshire HU5 5UN, UK; rabaillod@aol.com

**Keywords:** chronic fatigue syndrome, epidemic hysteria, mass hysteria, myalgic encephalomyelitis, psychosomatic illness, Royal Free epidemic

## Abstract

*Background and Objectives:* Controversy exists over whether myalgic encephalomyelitis/chronic fatigue syndrome (ME/CFS) is an organic disease or a psychosomatic illness. ME/CFS usually occurs as sporadic cases, but epidemics (outbreaks) have occurred worldwide. Myalgic encephalomyelitis was named to describe an outbreak affecting the lymphatic, muscular, and nervous systems that closed the Royal Free hospital for three months in 1955. Fifteen years later, two psychiatrists concluded that epidemic hysteria was the likely cause. ME/CFS research studies show multiple pathophysiological differences between patients and controls and a possible etiological role for infectious organisms, but the belief that ME/CFS is psychosomatic is widespread and has been specifically supported by the epidemic hysteria hypothesis for the Royal Free outbreak. Our objective was to obtain accounts from ex-Royal Free hospital staff who personally experienced the 1955 outbreak and evaluate evidence for it being an infectious illness versus epidemic hysteria. *Materials and Methods:* Statements in the newsletters of two organizations for staff who had worked at the Royal Free hospital invited anyone who had experienced the 1955 Royal Free outbreak to contact the authors. Accounts of the outbreak from telephone interviews and letters were evaluated against the “epidemic hysteria hypothesis” paper and original medical staff reports. *Results:* Twenty-seven ex-Royal Free hospital staff, including six who had developed ME, provided descriptions typical of an infectious illness affecting the lymphatic, muscular, and nervous systems, and were not consistent with epidemic hysteria. *Conclusions:* The 1955 Royal Free hospital epidemic of myalgic encephalomyelitis was an organic infectious disease, not psychogenic epidemic hysteria.

## 1. Introduction

Controversy exists over whether myalgic encephalomyelitis (ME), also known as chronic fatigue syndrome (CFS) and as ME/CFS, is an organic disease, a psychosomatic illness, or even exists as a disease entity. ME/CFS usually occurs as sporadic cases, but epidemics (outbreaks) have occurred worldwide [1,2]. In the summer of 1955, an illness, that had not been described in existing medical textbooks, affected more than 300 members of the medical, nursing, and ancillary staff at the Royal Free Group of hospitals in London [2,3,4]. The hospital medical staff reported that “this was an outbreak of an obscure, highly infectious illness with evidence of involvement of lymphoreticular structures and the central and peripheral nervous systems” and called it an encephalomyelitis [3,4]. The outbreak lasted from July to November and resulted in the main hospital being closed for three months. In spite of intensive investigation, no causal pathogen was identified [2,3,4]. No evidence was found that contaminated water, milk, or food was the source of infection and no toxins were found [3,4]. The illness was initially named Royal Free disease but the following year the name benign myalgic encephalomyelitis was coined to describe this and several other similar outbreaks [5]. The name chronic fatigue syndrome (CFS) was introduced in 1988 to describe a comparable disease, in Nevada, USA [6].

Fifteen years after the Royal Free outbreak, two psychiatrists (McEvedy CP, and Beard AW) published a hypothesis stating: “From a re-analysis of the case notes of patients with Royal Free disease, it is concluded that there is little evidence of an organic disease affecting the central nervous system and that epidemic hysteria is a much more likely explanation. The data which support this hypothesis are the high attack rate in females compared with males; the intensity of the malaise compared with the slight pyrexia; the presence of subjective features similar to those seen in a previous epidemic of hysterical over-breathing; the glove-and-stocking distribution of the anesthesia; and the normal findings in special investigations. Finally, a deliberate attempt by one of the authors to produce an electromyographic record similar to that reported in Royal Free disease was successful” [7]. They based their hypothesis on a study of 198 case notes selected from 255 hospitalized patients [3,7]. McEvedy and Beard also reviewed 14 other outbreaks identified as ME and proposed that they were psychosocial phenomena caused by mass hysteria on the part of the patients or altered medical perception of the community [8]. The concept of hysteria as the cause of the Royal Free outbreak was strongly opposed by the Royal Free medical staff on the grounds that there were characteristic physical signs, the disease was endemic in North London at the time of the outbreak, the disease course was prolonged, and epidemics had occurred worldwide [9,10]. 

The publication of the McEvedy and Beard papers ignited controversy over whether ME was an organic disease or a psychosomatic illness [2]. The following factors have been employed to support a psychosomatic hypothesis. The etiology is uncertain. There is no biomarker. Diagnostic criteria are based on clinical symptoms and the exclusion of other fatiguing illnesses. There are no pathognomonic physical signs. Patients frequently do not look ill even when severely affected by the disease. There is no curative medication. The concept that ME/CFS is a psychosomatic illness is widespread [11,12,13] and has resulted in the stigmatization of patients and patient complaints of sensing hostility from their health care providers [14,15].

Research studies in patients with ME/CFS have shown multiple pathophysiological differences between patients and healthy controls in the immune system, the nervous system, and metabolic processes including energy metabolism [16,17,18]. Although no causal pathogen has been identified, studies have shown that patients harbor a variety of infectious agents and have pointed towards a possible aetiological role for infectious organisms [19,20]. The psychosomatic hypothesis does not explain these pathophysiological changes. Mathematical modeling of the Royal Free outbreak also validates an infectious disease aetiology and refutes the epidemic hysteria hypothesis [21].

The question of hysteria or psychoneurosis as a possible cause was raised in the Royal Free outbreak [2,3], the 1934 Los Angeles county general hospital outbreak [22], and in three other outbreaks classified as ME [23]. Manifestations of psychoneurosis were seen in a few cases in all these outbreaks, but the authors concluded that hysteria did not explain the observed clinical features. Psychogenic anxiety reactions, evidenced by non-specific symptoms have been described in people exposed to outbreaks of organic disease, or people present during a disaster [24,25,26] and have been labeled “reactive psychological disaster syndrome” [26]. Reactive psychological disaster syndrome might account for some patients showing hysterical manifestations in various outbreaks of ME.

Our objective was to obtain first-hand observational accounts of the 1955 outbreak of ME from ex-Royal Free hospital staff and patients who had experienced it and to review evidence for the underlying cause being an organic infectious illness versus psychogenic epidemic hysteria. No other follow-up studies have been published. 

Etymology: ME/CFS has been labeled psychosomatic, psychosocial, somatoform, and a biopsychosocial illness. This paper uses the term psychosomatic. 

## 2. Materials and Methods

Statements were placed in the ‘Royal Free Association’ newsletter and the ‘Royal Free Nurses League’ magazine. These organizations were established for doctors and nurses respectively who trained or worked at the Royal Free Group of hospitals. The statements invited anyone who had experienced the Royal Free disease outbreak of 1955 to contact the authors to provide information about their experiences. We asked for information from both those who became ill and those who remained healthy. Volunteers contacted us by email and letters. Those who supplied a telephone number were interviewed using a semi-structured interview. Telephone participants were asked for their age and occupation at the time of the outbreak and were asked what they remembered about the event. Participants were included in the study if they had personally experienced the outbreak. The authors of this study were medical students at the Royal Free medical school and as such, were not permitted to enter the hospital at the time of the outbreak. Therefore, we did not meet the inclusion criteria for the study group. To avoid individual identification, descriptions of the outbreak in this paper are a compilation of individual accounts. Evidence for the outbreak being an infectious encephalomyelitis versus epidemic hysteria was evaluated by comparing the study group’s accounts and the original published medical staff reports [3,4] with data given for the epidemic hysteria hypothesis [7]. 

## 3. Results

### 3.1. Study Group

This study took place 58 years after the outbreak. Thirty people contacted the authors. Of these, 27 had personally experienced the outbreak and met inclusion criteria for the study group. Two responders provided information about friends who had developed Royal Free disease, and one told us about developing ME subsequent to the outbreak. Nineteen participants who provided a phone number were interviewed by phone. Participants’ data are given in Table 1.

### 3.2. Descriptions of the Outbreak

The study group confirmed that the outbreak started in July of 1955 and lasted several months. Importantly, staff in all five hospitals of the Royal Free Group were affected. People from the local community outside the hospital with symptoms of the disease were also seen in the casualty (Accident and Emergency) departments of the Royal Free and other London hospitals. The main Royal Free hospital in Gray’s Inn Road was closed to new admissions for three months due to a lack of healthy staff. Affected hospital staff were isolated at the Liverpool Road branch of the hospital, or were sent home. The epidemic was covered widely in national newspapers. The disease affected men and women, and both young and older, junior and senior staff. Very few existing hospital inpatients were affected. Most of those affected were nurses. Two study participants described secondary cases following close contact with a patient. The incubation period was “4–5 days” in one and “a few days” in the other. No secondary cases were reported following immediate visual exposure to a patient.

### 3.3. Descriptions of the Illness

Six study group participants developed the disease. Their experiences are compiled in Table 2.

The study group described a biphasic illness. Initially, there were prodromal symptoms and signs (see Table 2). Tender enlarged posterior cervical glands were a defining diagnostic feature. Initial symptoms persisted into the second phase of the illness. A few days after illness onset, diverse muscular and neurological manifestations developed in many patients. Muscular pain and tenderness occurred in the neck, back, and/or limbs. Reported neurological manifestations included ptosis, difficulty with focusing eyes, hemiparesis, mono-paresis, weakness of hand muscles, foot drop, various sensory losses, and hyperesthesia. Other reported symptoms included difficulty urinating, anorexia, nausea, and vomiting. Hyperventilation was not reported. Patients often delayed seeking medical care until several days after illness onset. Symptom severity ranged from mild to very severe. Myalgia was sometimes extreme, causing patients to cry with pain. A putative diagnosis of abortive Royal Free disease was proposed for patients with mild symptoms who lacked physical signs. The study group also reported that there were some patients lacking physical signs, who were thought to be neurotic or to have exaggerated their symptoms.

Many patients were diagnosed clinically without blood testing, but in patients who were tested, leukopenia, or lymphocytes typical of viral diseases were found. Leucocytes, characteristic of glandular fever (infectious mononucleosis) were not found. Paul Bunnell tests were negative except in a patient diagnosed with glandular fever. Cerebro-spinal fluid testing did not show changes typical of poliomyelitis. Electromyograms (EMGs) (carried out in some patients) showed unspecified findings regarded as characteristic of the disease. Possible causal pathogens were sought but none identified.

Treatment was symptomatic. Severe muscle pain sometimes required the strongest analgesics. Complete bed rest while symptoms lasted (except for walking to the toilet) was insisted on, followed by slow mobilization. Convalescence was advised for the same period of time as the duration of symptoms because an early return to work could precipitate a relapse. Patients were hospitalized for two weeks and upwards. A few very severely ill patients, some with paralyzses, were hospitalized for over six months and were widely investigated for many bizarre symptoms.

The time for recovery varied from a few days in those with the possible abortive disease to several weeks or months in others. Prolonged time to recovery also occurred in patients isolated at home. Some affected staff were only able to return to work part-time, and in several individuals, an unusual fatigue persisted for up to two to three years. Some patients appeared to recover but later relapsed. One patient committed suicide. Patients with persisting paralysis were transferred to a rehabilitation unit. A number of patients remained disabled and were unable to return to their previous occupations. We received a report of one patient in whom ME/CFS symptoms persisted long term.

Initially, a glandular fever (infectious mononucleosis)-like illness was diagnosed, but this was rejected because diverse neurological signs occurred and Paul Bunnell tests were negative. A poliomyelitis diagnosis was also rejected because muscle weakness clearly differed from the paralysis seen in poliomyelitis and lumbar puncture testing was inconclusive. The question of hysteria was raised as some patients were thought to be neurotic. However, since a large number of patients were seriously ill with significant physical signs, the study group indicated that most hospital staff believed that the outbreak was an infectious illness.

### 3.4. Long Term Health Effects Attributed to the Illness

We were told of five people who developed a persisting paralysis. They included one person each with ptosis, weakness of one hand, foot drop, wasting of hypothenar muscles of both hands, and severe weakness of one leg that required arthrodesis of the knee and ankle.

## 4. Discussion

Based on the recollections of all the 27 ex-Royal Free hospital staff and medical students who provided data for this study, hysteria as the underlying cause of the Royal Free outbreak seems inconceivable. Our study group’s accounts are based on their first-hand personal experiences. McEvedy and Beard based their epidemic hysteria hypothesis on an analysis of some selected patient case notes [7]. They did not provide any evidence from the follow-ups of patients who had had the disease or from hospital staff. Epidemic hysteria is a diagnosis of exclusion, but McEvedy and Beard provided no data to exclude an infectious disease as a cause of the outbreak.

### 4.1. Evidence for Infectious Illness

Although no causal pathogen was found in the Royal Free outbreak, epidemiological and clinical features were consistent with an outbreak of an infectious illness. The disease was present in the wider community of north London as well as in all five hospitals of the Royal Free group. It affected male and female, young and older staff. Case to case infection clearly showed an incubation period of several days and no immediate visual transmission. Initially, prodromal constitutional symptoms and upper respiratory signs of low-grade fever, pharyngitis, and cervical lymphadenopathy were present. After a few days, diverse muscular and neurological symptoms and signs appeared in many patients. Lymphocytes typical of viral infection were seen in some patients. The duration of the illness ranged from a few days to many months. Its severity ranged from patients with the possible abortive disease to patients with severe disease. The authors of this paper consider that the closure of a large teaching hospital in London for three months might be necessary to control an outbreak of a persisting, highly infectious disease, that affected a large number of hospital staff and that might be transmitted to hospital patients. On the other hand, an outbreak of epidemic hysteria would not be a sufficient cause to close a hospital.

### 4.2. Arguments for Epidemic Hysteria

McEvedy and Beard asserted that there was little evidence of organic disease affecting the central nervous system [7]. Our study group contradicted this assertion and reported diverse neurological manifestations in many patients and permanent paralysis in a few. The original hospital medical staff report describes 148 patients with involvement of cranial nerves and/or motor or sensory defects in the limbs and trunk [3]. The undoubted neurological manifestations in this outbreak are not found in epidemic hysteria.

Our study group confirmed that the majority of those affected were female nurses. In McEvedy and Beard’s cases selected for study, they found an attack rate of 0.8% in males and 11% in females and said this supported their epidemic hysteria hypothesis [7]. However, the original Royal Free hospital staff reports showed that females comprised 70% of the population at risk, and the attack rate was 10.4% for females and 2.8% for males [4]. These attack rates are comparable to those found in other outbreaks of ME, which ranged from 1.6% to 4% in males and 6.4% to 8.4% in females [23]. A high attack rate in females compared with males does not distinguish epidemic hysteria from ME.

McEvedy and Beard stated that the intensity of the malaise compared with the slight pyrexia supported their epidemic hysteria hypothesis [7]. Our study group confirmed malaise and mild pyrexia. Severe malaise that worsens with exertion is a cardinal feature of ME, but malaise and pyrexia are not features of mass hysteria [27,28]. Pyrexia is characteristic of infectious disease.

McEvedy and Beard noted the presence of subjective features similar to those seen in a previous epidemic (described by McEvedy [29]), of hysterical over-breathing in schoolgirls as evidence of epidemic hysteria. In this previous epidemic, many reported symptoms resembled the constitutional prodromal symptoms exhibited in the Royal Free outbreak. However, notably, hyperventilation was reported in 40% of the schoolgirls and tetany occurred in one-third of them [29]. Hyperventilation has been reported in 19%–32% of cases in outbreaks of mass hysteria [27,28]. McEvedy and Beard noted a raised respiratory rate only in four severely ill, Royal Free patients and speculated “this was a frightened and hysterical population whose over-breathing was intermittent and covert” [7]. Hyperventilation cannot be covert. Overt hyperventilation was not reported by our study group, nor reported in the original medical staff reports [3,4]. The notable absence of hyperventilation does not support the argument that the Royal Free outbreak resembled this outbreak of hysterical over-breathing in schoolgirls.

McEvedy and Beard noted “the glove-and-stocking distribution of the anesthesia” as evidence of epidemic hysteria and commented ”It seems fair to say that the characteristic pattern of sensory loss is a classically hysterical one” [7]. They found a glove-and-stocking type of anesthesia recorded in the charts of 13 patients, 11 of whom were also severely ill [7]. Our study group did not report details of sensory losses, but the original medical staff reports stated that “objective sensory loss was usually maximal peripherally, and frequently coincided with motor weakness” [3]. Glove-and-stocking anesthesia may occur in patients with a hysterical conversion disorder, but it can also be due to peripheral neuropathy in many serious organic diseases. This type of conversion disorder has not been reported in any published outbreaks of mass hysteria [27,28] and its presence in patients who are also seriously ill is questionable. A glove-and-stocking type of anesthesia in a few seriously ill patients does not support a diagnosis of epidemic hysteria.

Our study group said that EMG recordings of muscles affected by Royal Free disease showed characteristic features. To support their epidemic hysteria hypothesis, McEvedy and Beard stated that “a deliberate attempt by one of the authors to produce an electromyographic record similar to that reported in Royal Free disease was successful” [7], with the implication that abnormal EMG tracings of patients with Royal Free disease might have been fabricated. They published an EMG tracing of the extensor digitorum of the arm from a healthy person while encouraging the outstretched arm to tremble and suggested a similarity between this tracing and the EMG tracing of a weak tibialis anterior muscle affected by Royal Free disease during maximal sustained volition, that had been published by the Royal Free medical staff [4,7]. They proposed that the EMGs in the Royal Free patients could have been produced by “maximum effort” [7]. Whether an EMG of a healthy arm muscle, while encouraging the arm to tremble should be equated with an EMG of a weak leg muscle under maximal sustained volition from a patient suffering from Royal Free disease is questionable, but this attempt to imply that the experienced Royal Free medical staff might have misinterpreted EMG data or that one Royal Free patient might have fabricated an abnormal EMG does not provide evidence that the Royal Free outbreak was epidemic hysteria.

Distinguishing epidemic hysteria from an organic illness can be difficult, but characteristic features can help with diagnosis. In epidemic hysteria outbreaks, person-to-person i.e., visual transmission usually occurs within minutes, i.e., the [27,28]. Contrary to this, our study group reported an incubation period of several days. In epidemic hysteria, symptoms usually quickly resolve in patients separated from other patients and from the environment where the outbreak began [27,28]. In the Royal Free outbreak, patients sent home did not recover quickly. The incubation period and the failure of symptoms to resolve in isolated patients is not consistent with epidemic hysteria.

### 4.3. Reactive Psychogenic Symptoms

Diagnostic difficulties occurred in a minority of patients who were thought to be neurotic or to have exaggerated their symptoms. We suggest that at least some patients might have developed “reactive psychological disaster syndrome” [26] as a result of knowing that they had been exposed to a serious, debilitating, infectious disease of an unknown cause. A minority of patients with possible reactive psychogenic symptoms does not invalidate an organic cause for the outbreak.

### 4.4. SARS CoV 2

Recent reports show that some patients infected with SARS CoV 2 have developed post-viral symptoms characteristic of ME/CFS [30]. Given the growing recognition of similarities between ME/CFS and post-viral SARS CoV 2 [30], we hope that these patients are not regarded as having a psychosomatic illness. We also hope that future studies investigating features of both diseases may lead to new treatments that could potentially be of benefit for both groups of chronically ill patients.

### 4.5. Strengths and Limitations

The study group all experienced the Royal Free outbreak of ME as hospital staff, medical students, and some as patients. The outbreak was dramatic and the participants provided clear first-hand eye-witness accounts. The authors of this study were medical students at the Royal Free medical school at the time of the outbreak. Our recollections are consistent with the findings of this study. The study participants were self-selected members of two organizations for staff who worked or trained at the Royal Free hospital and may not be representative of the hospital staff at the time of the outbreak. This study took place 58 years after the outbreak and the participants’ recollected accounts are subject to recall bias, are dimmed by the passage of time, and lack specific details of clinical findings.

## 5. Conclusions

This study obtained new eye-witness accounts of the 1955 Royal Free outbreak of ME from ex-Royal Free hospital staff, medical students, and patients who had developed the disease. Clinical and epidemiological features described by them, are consistent with an outbreak of an infectious illness affecting the lymphatic, muscular, and nervous systems, with long-term neurological defects in a few cases. Their accounts did not describe the expected features of epidemic hysteria. McEvedy and Beard’s hypothesis that epidemic hysteria was the cause of this outbreak was based solely on the examination of selected patient case notes. We show that data given by McEvedy and Beard to support their epidemic hysteria hypothesis are flawed. Specifically, some data was contradicted by the study group’s first-hand accounts of the outbreak. Some data did not distinguish between epidemic hysteria and ME. Some data preferentially supported an organic etiology, and some data was of doubtful validity. This study confirms that ME/CFS is an organic disease and repudiates the hypothesis of it being a psychosomatic illness.

## Figures and Tables

**Table 1:** Study Group Participants.

Participants	Number
Age: 75–85 years	27
Gender	26 females, 1 male
Occupation in 1955	9 doctors, 5 nurses, 12 medical students, 1 physiotherapist
Royal Free disease diagnosis	19 remained healthy, 6 were diagnosed with Royal Free disease,2 had mild symptoms possibly, abortive Royal Free disease

**Table 2:** Patients who developed Royal Free disease.

Features of the Disease	Patient Experiences
Initial prodromal symptoms	Severe pressure headache, malaise, exhaustion, feeling weak, feeling hot, dizziness, feeling drowsy, hypersomnia, and sore throat
Initial physical signs	Fever, pharyngitis, enlarged cervical glands
New symptoms and signs manifested a few days after onset	Severe weakness in one or both legs causing difficulty in walking, painful muscular twitching or spasms, hemiparesis, difficulty focusing eyes
Testing	Blood testing not done routinely. EMG done on one patient *
Severity	Mild or moderate
Hospitalization	Two to five weeks
Treatment/ management	Complete bed rest while symptoms lasted, (except for walking to the toilet). Convalescence for the same length of time as illness duration
Length of illness	Two to three weeks to two to three months
Recovery and return to work	Return to work in one to six months. Recovery was often incomplete because easily tired. Some returned to work part-time
Relapse	Relapse after two months back at work
Long term effects	Unusual fatigue persisting for 2–3 years. A return of muscular twitching when under stress in later life. A muscle paralysis

* The electromyogram (EMG) showed changes that were associated with Royal Free disease.

## Data Availability

Enough data is given in the paper to show exactly how the was done. There is no further data.
